# The Open Source Brain Initiative: enabling collaborative modelling in computational neuroscience

**DOI:** 10.1186/1471-2202-13-S1-O7

**Published:** 2012-07-16

**Authors:** Padraig Gleeson, Eugenio Piasini, Sharon Crook, Robert Cannon, Volker Steuber, Dieter Jaeger, Sergio Solinas, Egidio D’Angelo, R Angus Silver

**Affiliations:** 1 Department of Neuroscience, Physiology and Pharmacology, University College London, London, UK; 2 School of Mathematical and Statistical Sciences, School of Life Sciences, Arizona State University, Arizona, USA; 3 Textensor Limited, Edinburgh, UK; 4 School of Computer Science, Science and Technology Research Institute, University of Hertfordshire, UK; 5 Department of Biology, Emory University, Atlanta, USA; 6 Department of Neuroscience, University of Pavia, Pavia, Italy

## 

While an increasing number of biophysically detailed neuronal models (featuring (semi-) realistic morphologies and voltage and ligand gated conductances) are being shared across the community through resources like ModelDB, these usually only represent a snapshot of the model at the time of publication, in a format specific to the original simulator used. Models are constantly evolving however, to take account of new experimental findings and to address new research questions, both by the original modellers, and by other researchers who help provide quality control/debugging of original scripts and convert the model (components) for use in other simulators. This crucial part of the model life cycle is not well addressed with currently available infrastructure.

The Open Source Brain (OSB) repository is being developed to provide a central location for researchers to collaboratively develop models which can be run across multiple simulators and can interact with the range of other applications in the NeuroML “ecosystem”. NeuroML [1] is a simulator independent language for expressing detailed single cell and network models, which is supported by an increasing number of applications for generating, visualising, simulating and analysing such models as well as by databases providing the base components (e.g. reconstructed morphologies, ion channels) for use in the models (http://www.neuroml.org/tool_support). The OSB repository differs from existing model databases which have traditionally concentrated on frozen, published models. The cell, ion channel, synapse and network models in this repository develop over time to ensure they reflect best practices in neurophysiological modelling and allow continuously improving, bug-free simulations. Multiple views of the model elements are available to encourage feedback from modellers, theoreticians and experimentalists. Links can be made to previous versions of the models in ModelDB, and deep links will be used to ensure cross referencing to other neuroinformatics resources such as NeuroMorpho and NeuroLex.

The system is based around a Mercurial version control repository with models organised into projects illustrating a number of neurophysiologically relevant aspects of the cell and network behaviour. The history is recorded of all changes to each project by contributors who can be distributed worldwide. There is close integration with the application neuroConstruct [2], allowing the models to be examined with a 3D graphical user interface, and scripts automatically generated for use on a number of widely used neuronal simulators. A number of models are already available in the repository, including cell and network models from the cerebellum, detailed cortical and hippocampal pyramidal cell models and a 3D version of a single column thalamocortical network model [3]. While most of the models available are conversions of existing published models, some have been developed during original research projects using the tools and formats discussed here [4]. The repository is currently in alpha stage of development and is being tested with a small number of labs. The resource can be accessed at http://opensourcebrain.org:8080. This work has been primarily funded by the Wellcome Trust (086699/095667).
